# EHPnet: EPA–China Environmental Law Initiative

**Published:** 2008-08

**Authors:** Erin E. Dooley

http://epa.gov/ogc/china/initiative_home.htm

China’s recent rapid economic expansion has created an array of serious environmental problems for that country, which Chinese officials are working to rectify with a solid environmental law framework. But implementing such laws is not always easy. In the fall of 2007, after a series of meetings with organizations in several Chinese cities, the U.S. Environmental Protection Agency (EPA) General Counsel launched the EPA–China Environmental Law Initiative to facilitate discussion on ways to foster environmental legislation and regulation. Information about this program is available online in both English and Chinese.

Visitors will find links to the websites of the initiative’s collaborators, all of whom have experience in Chinese environmental law and the environmental matters encountered by U.S. businesses operating in China. There is a Legal Resources page of information on Chinese environmental statutes, regulations, and directives as well as treaties and other international cooperation activities to which the country is party. Also on this page are links to recent reports and other publications pertinent to the scope of the initiative.

Recent relevant news articles are arranged on the homepage within subject headings that include pollution control, public participation, climate change/energy, and sustainability/pollution prevention. An archive of older items is also available. The homepage also features a calendar of upcoming events.

